# Modelling Alternative Economic Incentive Schemes for Semi-Natural Grassland Conservation in Estonia

**DOI:** 10.1007/s00267-024-02011-2

**Published:** 2024-08-01

**Authors:** Takamasa Nishizawa, Johannes Schuler, Claudia Bethwell, Michael Glemnitz, Maaria Semm, Monika Suškevičs, Laura Hämäläinen, Kalev Sepp, Rando Värnik, Sandra Uthes, Joachim Aurbacher, Peter Zander

**Affiliations:** 1 Leibniz Centre for Agricultural Landscape Research (ZALF), Müncheberg, Germany; 2 Geography Department, Humboldt-Universität zu Berlin, Unter den Linden 6, 10099 Berlin, Germany; 3 Estonian University of Life Sciences, Institute of Agricultural and Environmental Sciences, Chair of Environmental Protection and Landscape Management, Kreutzwaldi 5, 51006 Tartu, Estonia; 4 Estonian University of Life Sciences, Institute of Agriculture and Environment, Chair of Rural Economics, Kreutzwaldi 1, 51006 Tartu, Estonia; 5 Justus-Liebig-University Giessen, Institute of Farm and Agribusiness Management, Giessen, Germany

**Keywords:** Semi-natural grasslands, Abandonment, Sustainable rural development, Integrated land-use modelling, Economic viability, Habitat quality

## Abstract

Semi-natural grasslands (SNGLs) in Estonia are threatened by abandonment. This threat is leading to concerns about the degradation of biodiversity within grassland communities. Despite the high relevance of economic incentives in this context, how such incentives influence land managers’ decision-making regarding the agricultural use of SNGLs has not been investigated. To obtain its socio-ecological implications for policy-making, we developed regionally specific agricultural scenarios (compensation payments, livestock capacity, hey export, and bioenergy production) and an interdisciplinary modelling approach that made it possible to simulate agricultural land use changes through land managers' responses to varied economic conditions. Through this approach, we found that some economic factors hampered the use of SNGLs: the moderate profitability of beef production, labour shortages, and the relatively high profitability of mulching. We observed a positive relationship between SNGLs and habitat suitability for breeding and feeding birds. However, due to the high maintenance costs of SNGLs, the modelling results indicated that increasing the use of SNGLs through public budgets caused crowding-out effects, i.e., the deteriorating market integration of regional agriculture. This study emphasises the need for policy measures aimed at cost-effective, labour-efficient management practices for SNGLs.

## Introduction

Semi-natural grasslands (SNGLs) support a diverse range of species that have evolved to thrive under unique conditions (Brüggeshemke et al., 2022; Prangel et al., 2023). Across Europe, vast rural areas are covered by species-rich semi-natural habitats, including meadows and wooded pastures (Wilson et al., 2012). SNGLs in Estonia have exceptionally high levels of biodiversity, in particular wooded meadows (76 species/m^2^; Kukk (2004); Kukk and Kull (1997)), alvars (63 species/m^2^; Pärtel et al. (1999)), floodplain meadows (50 species/m^2^; Truus and Puusild (2009)), and coastal meadows (34 species/m2; Burnside et al. (2007)), among the most species-rich habitats in Northern Europe (Benstead et al., 1999). The benefits associated with the conservation of SNGLs extend beyond biodiversity; SNGLs are directly linked to various socio-economic factors in rural communities (Perpiña Castillo et al., 2021). For example, conservation efforts provide employment opportunities for agricultural production, contribute to the preservation of cultural heritage and traditional cultural landscapes, and support ecotourism activities (Veidemane, et al. 2019). This is why SNGLs are said to be a part of broader socio-ecological systems, contributing to the sustainable development of rural communities (Burnside et al., 2007; Pärtel & Helm, 2007; Sammul et al., 2008).

Despite increasing awareness of SNGLs as high nature-value grasslands, SNGLs in Estonia have been continuously declining since the 1950s (Kana et al., 2008). Between 1957 and 1960, 90% of coastal meadows were grazed or mown, whereas between 1992 and 1995 only 35% of coastal meadows remained in use (Kaisel et al., 2004). As of 2020, there were 130,000 hectares of SNGLs in Estonia, but the current area is estimated to be insufficient to secure the survival of the habitats of the protected species associated with these areas (Helm and Toussaint, 2020). Increasing evidence suggests that the abandonment of SNGLs leads to biodiversity loss within grassland communities (Kull & Zobel, 1991; Pärtel et al., 2005; Ward et al., 2013). Agricultural management, particularly, grassland-based extensive beef production through SNGLs, plays a crucial role in maintaining SNGLs (Eriksson, 2022; Directorate-General for Environment, 2023). Reintroducing traditional management practices is a recommended strategy (Valkó, et al., 2018) but it requires support through appropriate policies, such as regulations, subsidies, or innovative measures that aim to increase the demand for grassland products (Waldén and Lindborg, 2018). The economic viability of managing SNGLs is a highly relevant factor in this context (Kikas et al., 2016; Zindler et al., 2023). While there are some studies incorporating economic analysis into the evaluation of grassland conservation policies for their cost-effectiveness (Gerling et al., 2023; Robert et al., 2017; Wätzold et al., 2016), how it influences land managers’ decision-making, particularly focusing the agricultural use of SNGLs, has been less studied. Most studies on conservation measures for SNGLs have been concentrated in the agronomic or ecological fields (Waldén and Lindborg, 2018; Johansen et al., 2019; Villoslada Peciña et al., 2019; Herzon et al., 2021).

Therefore, this study aims to explore how the agricultural use of SNGLs can be promoted by examining the effects of various economic incentives for land managers, using a rural Estonian region as a case study. We assess the impacts of resulting land use changes through the land manager’s decision-making considering various factors crucial to sustainable rural development. To this end, we develop an interdisciplinary land use impact assessment framework, and integrated land use modelling. Within this modelling framework, agricultural scenarios are developed that describe alternative regionally specific economic incentives. These scenarios reflect the current regional strategies for increasing the demand for grass products. The bio-economic farm model (BEFM), in turn, simulates land use change via land managers’ decision-making under the scenario conditions. We estimate habitat quality on agricultural land by combining an ecological model with the BEFM to extend the assessment of land use changes to both ecological and socio-economic dimensions. Given that agriculture is closely tied to rural development, we assess the impacts of the agricultural scenarios based on three aspects: cultural heritage, rural economic profitability, and farmland biodiversity.

With this approach, we address the following research questions:

1. How will different economic conditions influence the agricultural use of SNGLs?

2. How will the change in grassland use affect rural economic profitability and farmland biodiversity?

3. What trade-offs are observed between these three considered aspects of sustainable rural development?

## Methodology and Data

### Case Study Region

**Fig. 1:**
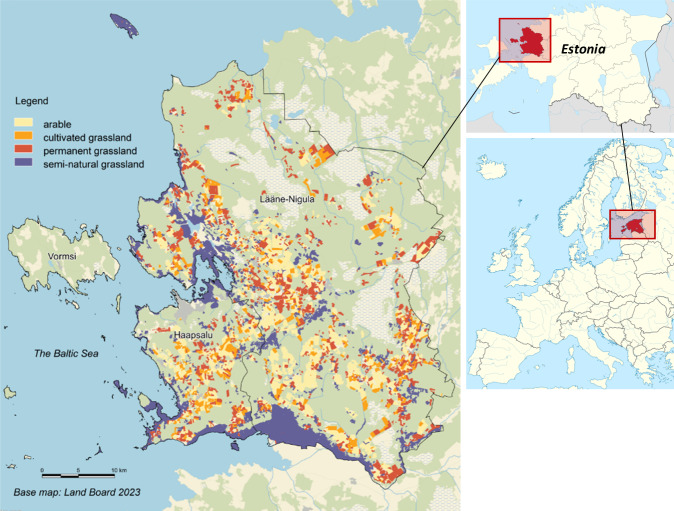
Land use classes in the case study region, Lääne County (Estonia) on the left (Source: the map layer of SNGL Estonian nature information system (EEA), the inventory of SNGLs of the Estonian Semi-Natural Community Conservation Association (ESCCA), and the Register of Agricultural Support and Agricultural Parcels (ARIB), and the map layer of SNGL maintenance support (ARIB). The maps on the right are distributed under a CC BY-SA 3.0 licence)

Our case study region is Lääne County (Fig. 1), a western coastal area of Estonia facing the Baltic Sea to the west. It consists of three municipalities: Lääne-Nigula, Haapsalu Town, and Vormsi (a small island in the far west of Lääne County). We excluded Vormsi from this study because the land on the island is rarely used for agriculture. The area is characterised as predominantly flat land. The average annual temperature is between 6.1 °C and 7.8 °C, with precipitation of 500–700 mm.

Based on the map layer of SNGL in the Estonian nature information system (2022) (Estonian Environment Agency/EEA), the map layer of the inventory of SNGLs of the Estonian Semi-Natural Community Conservation Association (2022) (Estonian Semi-Natural Community Conservation Association/ ESCCA) and the Register of Agricultural Support and Agricultural Parcels (2022) (Agricultural Register and Information Board/ARIB), and the map layer of SNGL maintenance support (ARIB, 2022), approximately 43,000 hectares is used for agriculture, 57% of which is arable land, 31% is SNGLs and 12% is more intensively used permanent grassland. In this study, we defined SNGLs as naturally grown grasslands without planting cultivated plant seeds, ploughing, or fertilising at a specific time, while permanent grasslands as grass fields without being interrupted by the cultivation of arable crops for at least 5 consecutive years. Fertilising is possible for permanent grassland. SNGLs fall under permanent grassland but require additional conditions that natural biota have been formed under the influence of long-term human activities (mowing, grazing), which are included in the range of habitats protected at the EU level. Approximately half of all SNGLs are estimated to be abandoned in Lääne County. We counted fields as abandoned that have not received agri-environmental payments (AEPs) or direct payments in the last 2 years. Over time, abandoned fields are typically covered by shrubs, reed beds, or forests.

The main crops on AL are field grass (referred to as cultivated grasslands in Estonia), winter wheat, spring barley, and winter rapeseed. Based on the map layer of the inventory (ARIB, 2022), the total number of farm holdings that own more than one hectare of land in Lääne County is 476 with an average farm size of 78 hectares. Most farm holdings are mixed farms with combinations of arable crops and animal husbandry (mostly beef cattle for meat production) or focused on crops. The region is suitable as an example because most SNGLs have been preserved in this area; thus, various conservation efforts have been made, including the restoration of abandoned SNGLs and the construction of a biomass heating plant to increase the demand for grass products.

### Integrated Land Use Modelling

**Fig. 2:**
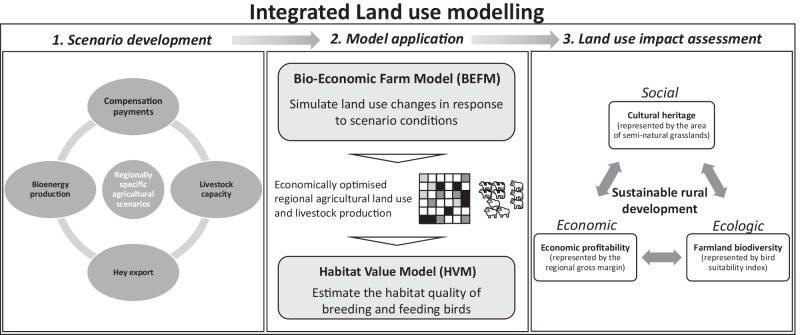
Framework of integrated land use modelling

Figure 2 illustrates the framework of the integrated land use modelling. The first part is the scenario development. The second part represents the integrated modelling application that combines the BEFM with the Habitat Value Model (HVM). The third and final part is the assessment of land use changes resulting from the scenarios, considering three aspects of sustainable rural development: cultural heritage for a social aspect (represented by the area of SNGLs), economic profitability for an economic aspect (represented by the regional gross margin (GM)), and farmland biodiversity for an ecological aspect (represented by bird suitability index). We first explain the applied models and then introduce the scenarios and the indicators for the assessment of sustainable rural development.

### Bio-Economic Farm Model (BEFM)

#### General approach

The aim of developing the BEFM was to simulate the change in agricultural land use in response to varying economic conditions based on the land managers' decision-making. Thus, we consulted earlier work on BEFM, such as the multi-objective decision support tool for agro-ecosystem management (MODAM) by Schuler and Kächele (2003), Schuler et al., (2013, 2020), and Uthes et al. (2010), as well as more recent studies by Nishizawa et al. (2022, 2023).

We first pooled 1-year data on 8689 agriculturally used fields in Lääne-Nigula and Haapsalu Town that were registered in ARIB (2022) to form “the regional farm” in accordance to Rounsevell et al. (2003) and Glemnitz et al. (2015). This regional farm represents the land-use decision-making of all land managers in the targeted areas and disposes of all available farm resources as one aggregated entity, given established farm structures, including total livestock, farmland, and farm labour. ARIB (2022) includes the field size, crop type and land use (permanent grassland or arable) of each field but no information on the grassland type (SNGLs, permanent grassland, or cultivated (field) grassland) or SNGL management practices (grazing, mowing, or abandoned). To obtain these missing data, a GIS-based dataset retrieved from the map layer of SNGL maintenance support (2022), a map layer of SNGLs in the Estonian nature information system (2022) and a map layer of the inventory of SNGLs of the Estonian Semi-natural Community Conservation Association (2022) was used. Cultivated grassland was distinguished by AL.

In the optimisation process, the total GM of the region is maximised, assuming that the regional farm engages in profit-maximising behaviour. Hence, regional agricultural land use is understood as the manifest of their aggregated land-use decisions. The optimisation algorithm follows the general form of linear programming (LP) for n activities and m structural restrictions:

1 $$\begin{array}{cc}{\rm{maximise}} & Z=\,\mathop{\sum }\limits_{j=1}^{n}{c}_{j}{x}_{j}\end{array}$$

2 $$\begin{array}{ccc}{\rm{subject\; to}} & \mathop{\sum }\limits_{j=1}^{n}{a}_{{ij}}{x}_{j}\le \,{b}_{i} & {\rm{for\; all}}\end{array}{i}=1,\,2,\ldots ,\,m$$

and

3 $$\begin{array}{cc}{x}_{j}\ge 0 & {\rm{for\; all}}\end{array}j=1,\,2,\ldots ,\,n$$

*Z* is the total GM of the regional farm, *x* represents the decision variables for the farm activities, *c* denotes the GM or cost per unit of activity, *a* is the technical coefficients, and *b* represents resource availability (land and labour) or the upper/lower limits of activities.

By maximising the regional GM, the economically optimal combination of crop activities and their area sizes, and, in our case, the optimal livestock numbers can be determined. Fixed costs, such as investments, paid labour, or rented land, were excluded from the optimisation, as the focus of this study was on short-term decision-making by the regional farm in response to changing economic conditions. Therefore, all the costs considered in LP are directly related to a production level (i.e. variable costs). The operation of LP was conducted with mathematical programming software (General Algebraic Modelling System—GAMS, version 31.2.0).

#### Modelled agricultural activities and constraints

##### Model activities

The following field activities were considered for the different site types (Table 1):

**Table 1:** Modelled field activities based on site types and livestock husbandry

SNGLs	Permanent grassland	Arable land	Livestock husbandry
• Grazing • Mowing • Biomass production for bioenergy • Mulching	• Grazing • Mowing (1 cut/year) • Mowing (2 cut/year) • Mowing (3 cut/year) • Mixed (hay-grazing) • Mixed (silage-grazing) • Mulching	• Field grass (hay and silage) • Mechanically tilled fallow • Field beans^a^ • Field peas^a^ • Green maize^a^ • Oats^a^ • Rye^a^ • Spring barley^a^ • Spring and winter wheat^a^ • Potatoes • Spring and winter rapeseed • Buckwheat^a^	• Dairy cows • Offspring of adult dairy cows • Beef cattle • Offspring of adult beef cattle • Sheep

*SNGLs* semi-natural grasslands

^a^arable crops with a possible choice of three different management options (tillage, reduced tillage, and direct seeding). Other crops are managed only through ploughing

SNGLs: grazing for beef cattle and sheep; mowing (1 cut) for hay and biomass production for generating heat (bioenergy); and mulching. Ploughing and fertilisation are not allowed. Biomass production for bioenergy is harvested only from SNGLs, and its demand is limited by the existing demand for biomass heating. Grass cut through mulching on SNGLs is collected from fields as a recommended practice for the environment to preserve the original soil characteristics (Janssens et al., 1998; Bakker and Berendse, 1999) but is usually disposed of due to its low quality for feed purposes (Lepmets 2015). The remaining fields, which are not used by any of these activities, are counted as abandoned fields. The initial restoration costs associated with restoring abandoned SNGLs were not considered in the BEFM. Given that different grass habitats provide different yields and nutritional values, we assumed that grazing was conducted on coastal meadows and mowing was carried out on alluvial meadows.

Permanent grassland: grazing for all livestock types; mowing for hay production (1–3 cuts); and “mixed” (mowing for hay or silage production and subsequent grazing on the same field). These activities must not be interrupted by a crop rotation scheme and managed with fertilisation but without ploughing or pesticides. Unlike mulching on SNGLs, grass cut through mulching on permanent grassland is left on the fields. Producing hay for export (e.g. to Saudi Arabia) is possible, and both SNGLs and permanent grassland can be utilised for that purpose. We assume that the export of hay is carried out on a contractual basis.

Arable land: field grass for baling or silage; mechanically tilled fallow; and twelve different crops (field beans, field peas, green maize, oats, rye, spring barley, spring wheat, winter wheat, potatoes, spring rapeseeds, winter rapeseeds, and buckwheat), which follow a crop rotation scheme (see Table 3). While potatoes and rapeseeds are managed only through ploughing, three different management options are available for the other crops: tillage, reduced tillage, and direct seeding. Buckwheat, potatoes, and winter and spring rapeseeds are modelled solely as cash crops, while the other crops can be used for sale or as feed. Organic farming is not considered because it is not widely practised in the region according to regional experts.

For livestock husbandry, we modelled dairy cows, beef cattle, their offspring, and sheep by referring to Nishizawa et al. (2022). The observed number of sheep was counted as one LU of beef cattle for every six sheep and added to the total number of beef cattle cows to determine the initial livestock capacity.

The GM per unit of activity was calculated as revenue = yield × producer price − variable cost + public payments. Producer prices, variable costs, and public payments were based on the average for the 2019–2021 period taken from the Estonian farm data handbook published by the Agricultural Research Centre in Estonia (ARC, 2021). This source differentiates variable costs over three levels of yields. We selected the yield level that best matched the average yield over the last 5 years (2017–2021) in the study region, as reported by Statistics Estonia (https://www.stat.ee). Different yield levels were not considered in this study as they play no significant role in the regional GM or habitat suitability values (Glemnitz et al., 2015). The variable costs included all costs involved in fieldwork steps, such as input use (seeds, fertilisers, and pesticides), machinery use, and transportation.

As the ARC (2021) provides no activities for SNGLs, we referred to Piirsalu et al. (2019) for yield information; their results are based on an empirical study in Estonia, whereas the variable costs were extrapolated from those of permanent grassland in ARC (2021). As most locations of SNGLs tend to be localised in marginalised areas such as coastal areas (see Fig. 1), we considered additional machinery and fuel costs (130 €/ha) for travel and transport to and from farmsteads to SNGLs based on expert judgements. The GMs of dairy cows and beef cattle assumed a dairy cow with 9000 kg of milk production per year, as reported by Statistics Estonia, and one beef cattle with 650 kg, as this is the standard size recorded in the ARC (2021).

We considered two types of public payments paid per hectare per year (see Table 3): i. AEPs include direct payments such as the SAPs for which all used agricultural fields are eligible, payments for so-called greening measures for maintaining permanent grassland and diversifying crops, and payments for following a crop rotation scheme Table 3; ii. compensation payments are paid additionally for SNGL activities on top of direct payments except for mulching.

Labour supply: The BEFM was also allowed to hire seasonal labour for harvesting up to the observed level (18,000 h) with a wage of 10 €/h (both from Statistics Estonia). As we could not obtain information on the share of the total agricultural labour units that were actually allocated to field and livestock activities, we derived this information from the observed land use, by multiplying hectares with the labour needed per field and livestock activity.

Labour requirement: Each fieldwork and type of livestock husbandry requires specific labour hours per hectare or LU. Due to a lack of data in Estonia, we referred to MLUK (2021), an agricultural handbook of Brandenburg in Germany, as it shares comparable conditions with its rich grassland region. To account for the specific conditions of the study area, we added 1.0 h/ha (based on expert knowledge) to the calculated labour requirement for the SNGLs activities. This adjustment reflects the increased labour intensity resulting from the isolated locations of SNGLs. Please note that this was introduced in addition to the extra machinery costs. Table 2 summarises the key parameters of the modelled grassland activities. All the parameters and their data sources are provided in supplementary material 1.

**Table 2:** Modelled activities for producing grass products

Site type	Activity for grass products	Yield (t/ha)	Price of hay (€/t)	Variable costs (€/ha)	AEPs (€/ha)	Compensation payments (€/ha)	Labour demand (h/ha)
SNGLs	Grazing	1.2	0	70 (+130)	160	150	1.9 (+1.0)
	Mowing^a^	1.9	70	131 (+130)	160	80	2.4 (+1.0)
	Bioenergy production^a^	1.9	70	131 (+130)	160	80	2.4 (+1.0)
	Mulching	0	0	53 (+130)	160	0	0.6 (+1.0)
Permanent	Grazing	3.0	0	161	160	0	2.7
Grassland	Mowing 1 cut/year^a^	3.4	70	188	160	0	2.4
	Mowing 2 cuts/year^a^	6.0	70	335	160	0	5.0
	Mowing 3 cuts/year^a^	9.7	70	539	160	0	6.5
	Mixed (hay-grazing)	5.1	0	254	160	0	3.7
	Mixed (silage-grazing)	6.4	0	222	160	0	4.0
	Mulching	0	0	53	160	0	0.6
Arable land	Field grass (silage)	5.3	0	286	210	0	4.1
	Field grass (silage/bale)	5.3	0	409	210	0	5.9

The price of hay is 70 €/ha only when hay is sold on the market; otherwise, it is 0 €/ha. Additional variable costs and labour for SNGL activities are presented separately with brackets

^a^The hay produced from these activities can be used for feeding livestock and/or for sale

##### Model constraints

The optimal agricultural production pattern had to meet the following restrictions: market conditions (producer prices, hay export contracts, demand for biomass heating plants); EU agricultural regulations for receiving AEPs (maintaining permanent grassland, crop diversification, and ecological focused area); crop rotations; and available resources (land and labour). Table 3 lists all the restrictions implemented in the BEFM in the baseline scenario, which provides a model output based on the observed values of the required parameters.

**Table 3:** Modelled constraints (regulations, conventional practices, and the reflection of reality) implemented in the baseline simulation

SNGLs	Permanent grassland	Arable land	Livestock husbandry
• Fixed share (31%) • Maximum biomass production for bioenergy (<1200 t = equivalent to one biomass heating plant) • Upper limit of exporting hay^a^ (<2500 t)	• Fixed share (12%) • The existing permanent grassland must be maintained • Upper limit of exporting hay^a^ (<2500 t)	• Fixed share (57%) • Crop diversification • Ecological measures (>5% of AL) • Crop rotation (refers to the observed crop share and consultation with experts in agronomy—cereals <60%, wheat <40%, green maize <40%, oats <4%, potatoes <25%, rapeseeds <25%, buckwheat <2%)	• Fixed stable capacity • Nutritional needs of net energy lactation, crude protein, crude fibre • Maximum intake of dry matter • The upper limit of grazing based on the standard feed ratio in the ARC (2021) • Grazing SNGLs only for beef cattle and sheep

^a^The production of hay for export (e.g. to Saudi Arabia) is possible both on SNGLs (semi-natural grassland)

The following assumptions were made. First, the observed allocation of land among the three site types—SNGLs, permanent grassland, and arable land—was assumed to be fixed, preventing any expansion/reduction of the existing levels. This fixed distribution aligns with current regulations in the study region, where the transformation of SNGLs into permanent grassland or arable land is infrequently observed and the conversion of permanent grassland into arable land is expressly prohibited under the European Agricultural Policy’s greening measure (CAP), with few exceptions.

Second, we assumed that the regional farm was obliged to follow all the constraints listed in Table 3 to receive AEPs. Field beans and peas were considered ecological measures, as growing legumes is a typical greening measure. Regarding crop diversification, we checked whether the number of arable crops chosen in the baseline simulation was close to the observed number of crops, rather than adding a constraint to the model.

Third, the maximum capacity (stable places) of livestock was fixed based on observed livestock levels. Thus the model determines the optimal LU within this range.

Fourth, all livestock animals must follow a specific dietary plan, which differs across animals. The minimum intake level of net energy for lactation, crude protein, and crude fibre must be satisfied, while intake of dry matter must not exceed certain levels. These values were calculated based on DLG (1997). The nutritional values of the feed were taken from Piirsalu et al. (2019) for grass on SNGLs and from FEEDBASE (Agroscope, et al., n.d.) for other kinds of feed. In addition, we assumed that all the necessary feed was supplied through the above-described field activities, i.e. buying feed was not considered, which reflects regional practices according to regional experts.

##### Validation of the parameters

We validated the initial parameters used for the baseline scenario as follows. First, we ensured that the deviation of the land use pattern in the baseline simulation regarding crop choice and area size from the observed land use pattern was minimised. Second, we ensured that the determined livestock number reached the maximal livestock capacity, which is equal to the observed level. Third, we checked whether the ratio of the ingredients of feed determined in the BEFM (grazing, hay, silage, and concentrates) matched the standard feed ratio recommended by the ARC (2021) as much as possible.

### Ecological Modelling of the Habitat Quality of Agricultural Land Use

#### General approach

The ecological part of the integrated land use modelling was carried out using the HVM (Brandt and Glemnitz 2014, Glemnitz et al. 2015). This model can be used to determine the habitat quality of agricultural land for indicator bird species. The habitat quality for these indicator bird species is suitable for reflecting the biodiversity of agricultural land, both on arable land and grassland (Brandt and Glemnitz 2014) and thus can be used to assess the impact of the different incentive schemes on the habitat quality of arable land and grassland. The HVM differentiates between two types of habitat use of the bird species on grassland: 1.) breeding habitat and 2.) feeding habitat and overlays the habitat preferences of the indicator species with the potential habitat suitability of each agricultural land use activity. Based on these activities and their respective crop shares the results can be up-scaled to the regional scale per applied scenario (Glemnitz et al., 2015). Two indices were derived per indicator species the breeding habitat index, which expresses the relative number of successful breeds per species and year and the feeding habitat index, which expresses suitable habitat conditions for feeding. The index values for single species were aggregated for some interpretations.

#### Assessing the habitat preferences of the bird indicator species

On the one hand, information on habitat requirements was used for the modelling, whereby an adjustment was made to the bird requirements of the species in the study region for the five bird indicator species for arable land by regional bird experts: skylark (*Alauda arvensis*), lapwing (*Vanellus vanellus*), whinchat (*Saxicola rubetra*), red-backed shrike (*Lanius collurio*), and yellowhammer (*Emberiza citrinella*). The two grassland species, corncrake (*Crex crex*) and grey partridge (*Perdix perdix*), were specifically selected to cover the regional bird species occurrences in Estonia on grasslands and their habitat requirements were newly compiled by expert parametrisation according to Brandt and Glemnitz (2014).

#### Assessing the potential habitat suitability of the agricultural land use activities

**Fig. 3:**
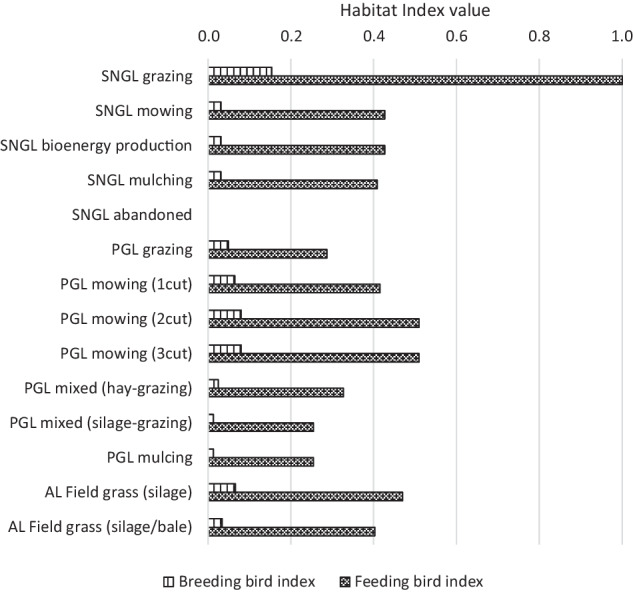
Habitat values of particular grass activities for breeding and feeding farmland birds. SNGL means semi-natural grassland. PGL permanent grassland. AL arable land

Over all 14 land use systems on grasslands including abandoned SNGLs and 29 land use systems on arable land, the potential habitat suitability was assessed. The vegetation structure was provided over the growing season by the particular land use systems, the cultivation period of the crops or grassland vegetation in 10-day periods, and necessary agricultural measures were compiled (Brandt and Glemnitz 2014). To do so, the definitions for breeding habitat suitability and feeding habitat suitability by Brandt and Glemnitz (2014) were used. The individual species' values estimated with HVM in this study were combined to create average values. Then the highest value of all the values calculated for both breeding and feeding birds was adjusted to 1.0 and any other values were scaled to this highest score. Figure 3 presents the adjusted habitat index values of land use systems on grassland. All the results from HVM including land use systems on arable land are provided in the supplementary material 2. The indices can be broken down to single species if needed to deliver assessments on the impacts on e.g. conservation foci on single species.

#### Matching the habitat preferences with potential habitat suitability agricultural activities and up-scaling

The bird habitat preferences of each bird species were matched with the provision of potential habitat qualities of each land use system (Brandt and Glemnitz 2014). The habitat suitability for breeding and the habitat suitability for feeding were summarised for the contributing bird species and analysed separately (Glemnitz et al., 2015). The regional habitat quality was subsequently assessed by combining the summarised breeding habitat values and the summarised feeding habitat values of each agricultural activity with the respective crop shares of these activities in the region per applied scenario (Glemnitz et al., 2015).

### Scenario Development

We developed a baseline scenario and four regionally relevant agricultural policy scenarios that described different economic conditions for grassland use as follows.

- Baseline scenario (simulated existing conditions): The land use simulated with the BEFM in this scenario replicates the existing regional agricultural land use, which is used for comparison in the following policy scenarios. Therefore, all the parameterisations in this scenario are based on the current data, which reflect the present land use.
- Scenario 1 (compensation payment for SNGL maintenance): Compensation payments are paid for maintaining SNGLs. We varied the level of payments between 0 and 200% of the current payment level. The payment levels for AEPs remained unchanged.
- Scenario 2 (higher livestock numbers of beef cattle based on premium meat brands produced on SNGLs): This scenario assumes a national marketing strategy for beef produced on SNGLs. We assumed higher livestock numbers in the region by subsidies for stable buildings, which we modelled as changes in the maximum capacity for beef cattle from 0 to 5000 LUs. The current total LUs of beef cattle in the region is 3200 LUs.
- Scenario 3 (increase in other countries’ demand for hay): This scenario reflects the current agricultural strategy of Estonia to increase the demand for grass products. We changed the export demand for hay from 0 to 5000 t. The current demand is 2500 t. In this option, the BEFM can choose the location of hay production (SNGLs and/or permanent grassland).
- Scenario 4 (bioenergy production—construction of biomass heating plants by regional heat suppliers): This scenario reflects the ongoing effort to search for alternative uses of grass on SNGLs. Currently, one biomass heating plant operates with a biomass demand of 1200 t of hay produced on SNGLs. According to the current regulations, only hay produced on SNGLs can be supplied to the heating plant. Therefore, unlike in Scenario 3, the choice of grassland in the BEFM was restricted to SNGLs. We changed the demand for such plants for hay from 0 to 4800 t.

### Land Use Impact Assessment

The scenarios were assessed with the above-explained applied models in terms of economic and ecological consequences, using the following three sets of indicators:

1. (Social) the share of used SNGLs to the total available SNGLs for agriculture, as a proxy for maintaining cultural heritage
2. (Economic) the regional GM and the market-generated GM of the region, as a proxy for rural economic profitability
3. (Ecologic) the two habitat quality values (habitat suitability for breeding and habitat suitability for feeding) of the region, as a proxy for farmland biodiversity

The used SNGLs were calculated by subtracting the simulated unused (abandoned) SNGLs from the total available SNGLs for agriculture. The regional GM, as maximised by the BEFM, can be an indicator of the overall income level of farm holdings in the region. The market-generated GM was calculated by subtracting all public payments from the regional GM; thus this metric reflects the ability of a region to continue to make profits in domestic and international markets, i.e. the market integration of regional agriculture. The habitat quality value of the region was calculated by multiplying the area of each farm activity by the corresponding habitat index value and dividing it by the total area of the region, given the assumption of a linear relationship between the habitat quality value and its area. All of these indicators were calculated at the regional scale.

## Results

### Baseline Land Use Pattern

In the baseline scenario, 58% of the SNGLs are used for agricultural production, most of which are used for grazing, while 42% of the available SNGLs are abandoned (unused) (Table 4). On permanent grassland, mulching is the dominant use (86%) (Table 4). The chosen arable crops are presented with management options. Winter wheat (reduced tillage), winter rapeseeds (ploughing), and spring barley (direct seeding) account for 80% of the arable land (Table 4). Field beans were grown on 5% of the arable land.

**Table 4:** Simulated land use patterns in the baseline scenario within each site type

SNGLs	ha	%	Permanent grassland	ha	%	Arable land	ha	%
Grazing	5328	43%	Mulching	7298	86%	Winter wheat/reduced tillage	7757	40%
Abandoned area	5219	42%	Mowing/1 cut	611	7%	Winter rapeseeds/ploughing	4848	25%
Mowing/1cut	1346	10%	Mixed (silage-grazing)	559	%	Spring barley/direct seeding	2909	1%
Bioenergy production	632	5%				Field grass-silage	1763	9%
						Field beans/direct seeding	970	5%
						Spring barley/reduced tillage	757	4%
						Buckwheat/reduced tillage	388	2%
Total	12,524	100%	Total	8468	100%	Total	19,392	10%

*SNGLs* semi-natural grasslands. (Source: own calculations)

### How Will Different Economic Conditions Influence the Agricultural Use of Semi-natural Grasslands?

**Fig. 4:**
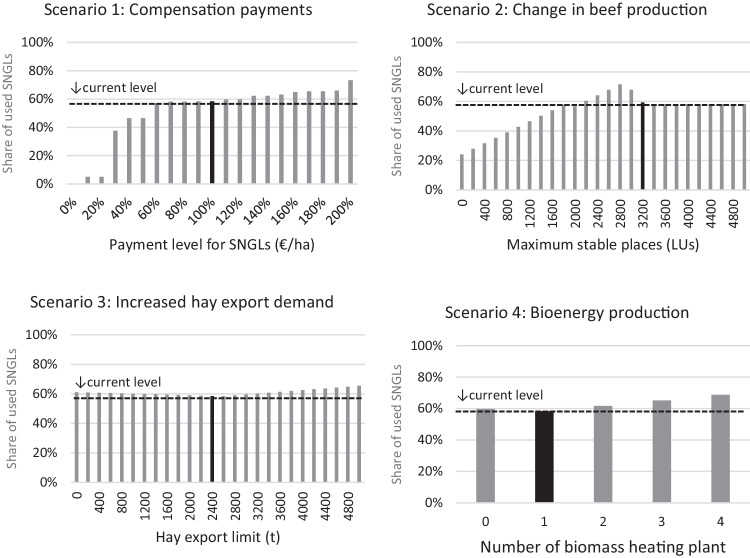
Changes in the share of used SNGLs (semi-natural grasslands) to the total available SNGLs for agriculture in the study region under different scenarios. The black-filled bars indicate the current conditions, that is, the simulated baseline land use pattern. The horizontal dotted lines indicate the level of SNGL use in the baseline simulation (58%). LUs mean livestock units. (Source: own calculations)

In scenario 1 (compensation payments, top-left in Fig. 4), the model showed a typical reaction to a targeted payment. The results changed stepwise until the next profitable level was reached, and then another activity was replaced by the subsidised activity. In particular, a payment level between 0% and 60% of the current level led to a sharp increase towards current levels of the SNGLs (from 0% to 57%, Fig. 4). However, no clear changes can be seen between 70% and 180% of the payment level (Fig. 4).

Scenario 2 (higher livestock numbers, top-right in Fig. 4) resulted in rather complex changes. With an increasing number of beef cattle, the share of used SNGLs continuously increased to a peak of 72% at 2800 LUs and then declined until a level of 3400 LUs. No further change was observed beyond that level. This finding indicates that it was not profitable for the model to further increase the number of beef cattle above 3400 LUs.

In scenario 3 (increased hay export demand, bottom-left in Fig. 4), two patterns of change could be observed. The used SNGLs declined until the 3000 t hay demand limit was reached but then started to increase beyond that level. This temporal decline of SNGL use is associated with the model behaviour for seeking efficient fodder production; hay production for export was carried out on SNGLs due to higher profitability but a part of fodder production shifted from SNGLs to permanent grassland to compensate for the increased demand for labour used on SNGLs. Therefore, the overall use of SNGLs declined. Increasing demand for hay beyond 3000 t was realised by reducing the number of beef cattle to bypass a labour shortage. Therefore, fodder production was shifted back to SNGLs as a reduction in beef production saved labour resources.

In scenario 4 (construction of biomass heating plant, bottom-right in Fig. 4), a pattern of change similar to that in scenario 3 was observed. The reason for this model behaviour was that hay production for export and grass for the heating plant were both produced on SNGLs due to relatively high profitability, although in scenario 3, hay production could have originated from either SNGLs or permanent grassland. Since the other conditions are equal in scenarios 3 and 4, the results show the same pattern of changes.

### How Will A Change in Grassland Use Affect Rural Economic Profitability and Farmland Biodiversity?

**Fig. 5:**
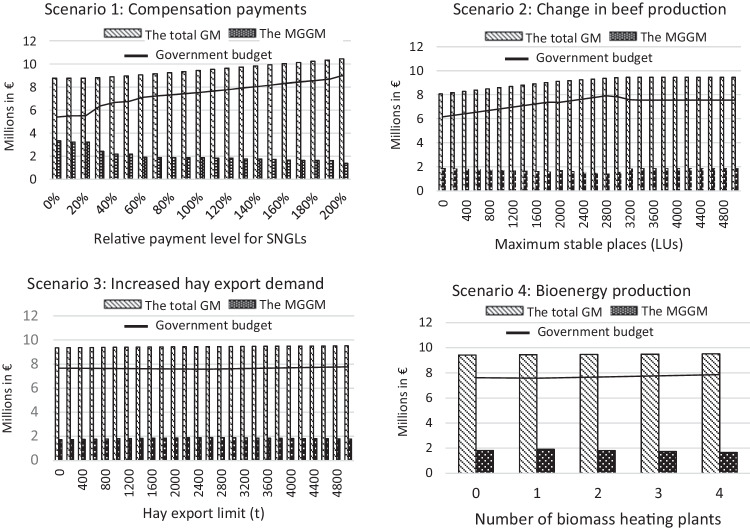
Change in the regional GM (gross margin) and the MGGM (market-generated gross margin) of the study region over the different scenarios. LUs mean livestock units. (Source: own calculations)

Figure 5 shows the changes in the regional GM and the market-generated GM in the study region across the different scenarios. The difference between them indicates the total amount of government budget (public payments) paid to the region, which is indicated with a line in Fig. 5. Relatively large effects on the regional GM and the market-generated GM can be observed in Scenarios 1 and 2 (Fig. 5). In scenario 1, the gap between the regional and the market generated GM became increasingly wider, which indicate an increasing government budget. The changes in the regional GM and market-generated GM exhibit comparable tendencies across all the scenarios; the change in the regional GM corresponds to the change in the used SNGLs. Due to the compensation payments for SNGLs, an increase in SNGLs led to an increase in the regional GM. In contrast, the market-generated GM decreased as the use of SNGLs and the regional GM increased. Nonetheless, as the land use decision in the BEFM is based on the regional GM, including public payments, the model first favours the use of SNGLs, even though it results in a reduction in the market-generated GM.

**Fig. 6:**
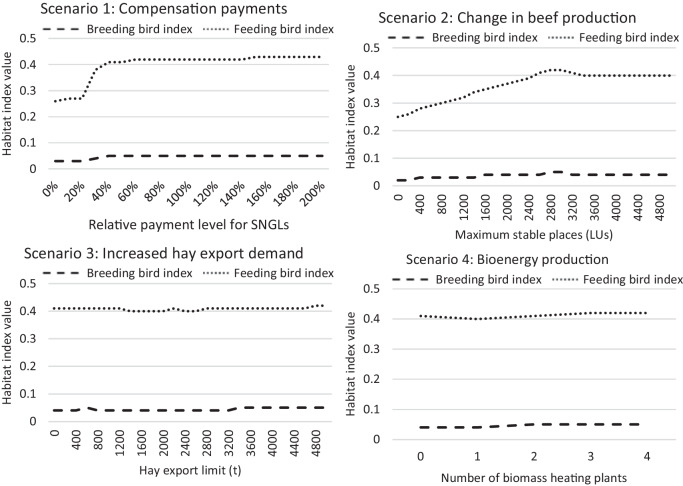
Change in habitat index values for breeding and feeding farmland birds across different scenarios. SNGLs means semi-natural grasslands. LUs mean livestock units. (Source: own calculations)

Figure 6 shows the changes in the habitat index values for breeding and feeding farmland birds across the scenarios. Little impact can be seen in the breeding habitat index values because the differences in the vegetation structure and the intensity of management activities among the land use systems on grasslands were not large (Fig. 3). In contrast, the feeding habitat quality for birds greatly changed in scenarios 1 and 2 (top-left and top-right in Fig. 6). This relatively large change is due to the high value of feeding birds for grazing on SNGLs compared to the other large values (Fig. 3). Therefore, if the use of SNGL changes significantly, the value for feeding birds will also change significantly.

### What Trade-offs Are Observed between the Examined Indicators?

**Fig. 7:**
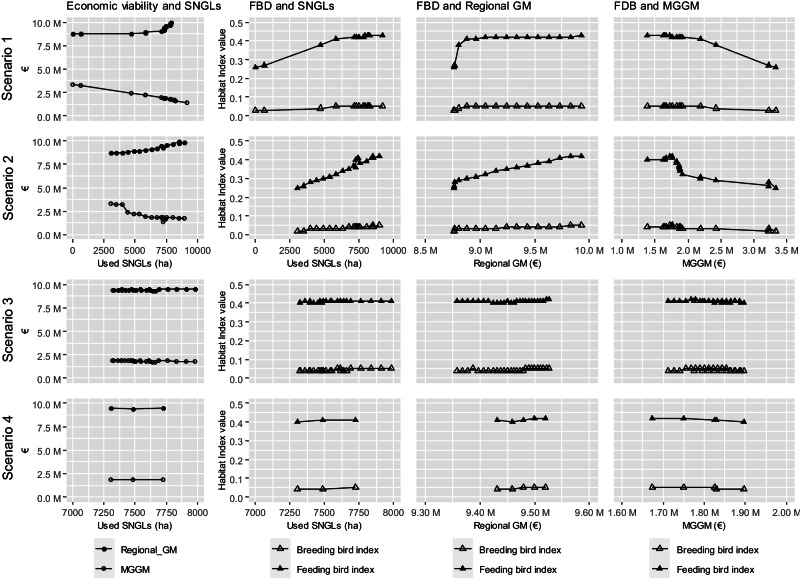
Relationship between the values of used SNGLs (semi-natural grasslands) (ha), the regional GM (gross margin) (€), the MGGM (market generated gross margin) (€), and FBD (farmland biodiversity) across scenarios (the results of scenario 1–4 are arranged in rows from the top to the bottom). (Source: own calculations)

Figure 7 plots the values of all the indicators (used SNGLs, regional GM, market-generated GM, and breeding and feeding bird indexes) calculated for the scenarios to show their relationships. The results are arranged in rows from scenarios 1–4, going from the top to the bottom. A similar tendency can be observed for scenarios 1 and 2 (Fig. 7, the top and second rows, respectively). The regional GM increased as the agriculturally used SNGLs expanded. In scenario 1, the use of SNGLs was driven by increased compensation payments, and in scenario 2, by increasing livestock numbers. However, the market-generated GMs in scenarios 1 and 2 both declined. The relative change of the market-generated GM is larger compared to that of the regional GM. Similar relationships were found for farmland biodiversity; farmland biodiversity and the used SNGLs are positively related. In scenarios 3 and 4 (Fig. 7, the third and fourth row), the values of the examined indicators showed no drastic change, as these scenarios had only moderate impacts on the use of SNGLs (Fig. 4).

## Discussion

### Interpretation of the Results

#### Economic conditions influencing the use of semi-natural grasslands

The baseline results imply that a substantial portion of permanent grassland would be left over for mulching, in case grasslands were used efficiently for feeding purposes. This result is influenced by the lower livestock numbers in the region relative to the area size: 1280 dairy cows, 2760 beef cattle, and 2772 sheep (ARIB, 2022). Mulching neither contributes to fodder nor hay production and is chosen solely as it is a minimum requirement for the payout of AEPs. This aligns with the actual strategy of land managers in the region, as confirmed through personal communication. The prevalent use of permanent grassland for mulching suggests that fodder production on SNGLs remains more profitable than that on permanent grassland due to compensation payments favouring SNGLs. However, these payments conceal the higher production costs on SNGLs, leading to an inefficient allocation of farm labour to SNGLs, despite the lower productivity.

The policy scenarios involving changes in compensation payments (scenario 1) and livestock capacity (scenario 2) had greater effects on the use of SNGLs than the scenarios involving changes in export limits (scenario 3) and bioenergy production (scenario 4). Overall, the results imply that the current level of compensation payments offers sufficient financial incentives for land managers to manage SNGLs. However, increasing public budgets in scenario 1 beyond the current level had only limited effects because of unchanged relative profitability among activities, whereas in scenario 2 the area used for SNGLs showed complex changes. Several economic factors accounted for this finding. As Fig. 4 shows, the first choice for fodder production for feeding beef cattle of the model was SNGLs, as it is more profitable to produce fodder on SNGLs. However, labour became a limiting factor at the peak level (2800 LUs); thus, some fodder production shifted from SNGLs to less labour-intensive activities on the permanent grassland to enable a further increase in the number of cows and earn more profits. A farm labour shortage in the study region was indeed confirmed by Kikas et al. (2016). In addition, the finding that unchanged land use further beyond a level of 3400 LUs indicates a labour shortage and only moderate GMs for beef cattle; compared to having more cattle, a higher GM could be obtained by allocating labour to the rather profitable mulching. This result is in line with Mõtus et al. (2017) and was also observed in Europe in general (Lherm et al., 2017; Manevska-Tasevska et al., 2014). This situation is attributed to the high production costs (e.g. labour, energy, and inputs) of European farming (Hocquette et al., 2018). The effects of moderate GMs on beef-fattening cattle also became apparent in scenario 3 (Fig. 4). Because hay production for export is more profitable than beef production, a higher demand for hay led to a reduction in the number of beef-fattening cattle to continue to produce hay for export on SNGLs. Similar to the case of mulching, hay production on SNGLs for export and biomass production for heat generates more profit than maintaining beef-fattening cattle. Therefore, the lower demand for feed shifted fodder production back to SNGLs and led to an increase in the used SNGLs.

#### The impacts of the use of semi-natural grasslands on economic profitability

The comparatively high impacts on the use of SNGLs in scenarios 1 and 2 resulted in considerable changes in the regional economic profitability, contributing to the overall income level of farm holdings in the region. When the compensation payments were abolished (to a level of 0%), there was a significant impact on SNGLs; while all the areas of SNGLs were abandoned, the market-generated GM reached the highest level among all scenarios. A similar increase in production through sacrificing biodiversity-rich land use was also found by Guillem et al. (2015) and Nishizawa et al. (2023). However, a further increase in the compensation payments beyond 60% in scenario 1 caused increasing windfall profits for landowners: the government budget continued to increase without clear effects on the use of SNGLs. A negative effect was also observed on the market-generated GM. Due to the higher production costs of managing SNGLs compared to those of permanent grassland, increasing the use of SNGLs pushed overall production costs higher. Therefore, increasing public budgets caused crowding-out effects and obstructed the market integration of regional agriculture. Crowding-out effects are generally referred to as situations where increased government spending leads to a decrease in spending in the private sector. This trade-off was more apparent in scenario 1 than in scenario 2, indicating that solely relying on compensation payments can weaken the ability of the agriculture sector to generate profits.

#### The impacts of the use of semi-natural grasslands on farmland biodiversity

Regarding farmland biodiversity, used grasslands are more important as feeding habitats for farmland birds than for breeding. All the scenarios showed a stronger impact on feeding habitat quality than on breeding habitat quality. An increase in the feeding habitat quality for birds was brought about by an increase in public budget dependency. The quality of grasslands used as feeding habitat for birds was highly sensitive to the use of SNGLs, as the change in their feeding habitat index value was nearly identical to the change in the use of SNGLs. This result means that returning SNGLs to extensive land use has beneficial effects on their role as feeding habitats for typical farmland birds. This finding is in accordance with Buckingham et al. (2006), who differentiated two groups of feeding behaviour and preferences: Species feeding on soil-dwelling invertebrates prefer short swards, while species feeding on sward-dwelling invertebrates or seeds prefer heterogeneous swards. A study by Katayama et al. (2015) revealed that the richness and abundance of agricultural wetland species in summer were negatively associated with both intensification and abandonment. Abandonment of grassland use on SNGLs might support breeding habitat suitability, e.g. wet grassland specialist bird species (Hanioka et al., 2018). Nevertheless, a comprehensive literature overview by Elliott et al. (2023) concluded that maintaining grassland management is crucial for supporting biodiversity conservation, including birds in European grasslands.

### Methodological Limitations

Due to the limited data and inherent complexity of SNGLs, several factors had to be simplified. First, only two types of habitats (coastal and alluvial meadow) could be considered in the BEFM. Even though they were the main habitats in the study region (53%) (ARIB, 2022), there are various habitats in the study region, including wooded pasture, wooded meadows, alkaline fens, and calcareous grasslands. The yields and nutritional levels and the compensation payments are also differentiated across different habitat groups in Estonia, all of which affect the productivity and profitability of grassland-based livestock production (Loucougaray et al., 2015). Second, we implicitly assumed that the restoration of SNGLs was possible regardless of site conditions and restoration costs. In reality, the efforts to restore SNGLs are affected by the extent of abandonment at the site (Joyce, 2014); the longer the fields have remained unused, the more challenging their restoration becomes. This reality indicates varied restoration costs and labour requirements. The different habitats of SNGLs and the site conditions are relevant not only to land use decision-making but also to the quality of farmland biodiversity. Many studies show the importance of incorporating landscape approaches into SNGL conservation efforts for biodiversity, in which the species composition and heterogeneity or configuration are taken into account (Harlio et al., 2019; Brüggeshemke et al., 2022).

In addition, some limitations of the approach employed for land-use modelling in this study should be noted. The tendency of LP models to overspecialise and produce jumpy solutions, particularly in a regional farm approach, may lead to more extreme land use patterns that would not occur in reality. However, it is a tolerable approach, given the lack of spatially explicit farm data and a relatively small case study area. Such issues can be handled by incorporating as many realistic land use restrictions as possible, including regulatory, agronomic, and biophysical conditions (in this study, the location of SNGLs) and by calibrating the model so that outcomes closely match the actual land use pattern, as explained in 2.3.2 Modelled agricultural activities and constraints.

Lastly, future studies should capture the long-term impacts of economic conditions on more extensive indicators relevant to sustainable rural development, such as biophysical conditions (e.g. soil quality or water availability) to consider their sustainability and multiple species groups to avoid over-interpretation of single species effects (Gossner et al., 2016; Gregory et al., 2019). Even though the scope of this study was the evaluation of the impacts of short-term land-use decisions, incorporating dynamic impacts into land-use modelling for a more comprehensive assessment would enhance the relevance and applicability of findings for long-term adaptive policy measures. This can be done by building feedback loop structures that enable the consequences of changing land use to be fed back into decision-making (Paul et al., 2019; Rodriguez-Gonzalez et al., 2020).

### Implications for Policy-making

Our findings show that compensation payments and livestock capacity can play a significant role in enhancing regional economic profitability and farmland biodiversity in marginalised agricultural areas. However, increasing compensation payments and livestock capacity from the current level resulted in a limited effect. This phenomenon causes, on the one hand, increasing windfall profits, an inefficient allocation of labour and a lower market-generated GM, on the other hand, crowding-out effects. To enhance the continued management of SNGLs for agriculture, increased demand for grassland products is essential (Waldén and Lindborg, 2018). In particular, grassland-based beef production plays the most important role (Nitsch et al., 2012; Bengtsson et al., 2019). However, since its profitability is not high and labour is scarce, fodder production is easily replaced with mulching or just aimed at exporting hay. Currently, compensation payments for SNGL are not tied to beef production, so one approach could include linking them to the maintenance of a minimum livestock density per unit area of grassland. Such payment schemes would incentivise land managers to maintain or increase livestock numbers and revitalise livestock production in the country. As a market-based solution, establishing premium brand beef that is fed on SNGLs might be a promising effort not only to secure the economic viability of beef production in the long term (Hocquette et al., 2018) but also to safeguard species-rich habitats (Magda et al., 2015). This trend has been gradually developed in the study region as consumer preferences for “locally” and “ecologically” produced agricultural products are increasing (Feldmann and Hamm, 2015; Rytkönen et al., 2018). A support scheme for grassland-based production that pays out land managers for positive effects on carbon sequestration could be also an option in the future in Estonia (Hall, 2018).

Another highlighted finding is that labour availability plays a decisive role in determining the use of SNGLs. As Kikas et al. (2016) found, the older age of farmers and lack of successors are strongly related to abandonment. As shown in this study, managing SNGLs is labour-intensive due to their isolated locations. Even though compensation payments help increase the use of SNGLs, farm labour, which is already a scarce resource in the region, will be consequently more inefficiently allocated. Therefore, conservation measures for SNGLs should not focus solely on reintroducing traditional management practices (Valkó, et al., 2018), which are not economically viable or profitable; rather, the implementation of cost-effective management practices to reducing labour requirements is the key to successfully restoring SNGLs. This could involve strategies, such as utilising more efficient machines or establishing specialised service providers, particularly if the profitability is high enough. As the factors that hinder the use of SNGLs might change depending on local conditions, such strategies should be adapted to local conditions (Plieninger et al., 2016).

## Conclusions

Various economic conditions can substantially influence all aspects of sustainable rural development in a marginalised agricultural region. The use of SNGL is closely related to rural economic profitability and farmland biodiversity. Our findings imply that the current level of compensation payments for SNGL management provides land managers with sufficient financial incentives, and increasing the use of SNGLs is likely to have large positive effects on habitat quality, especially for feeding birds. Among the scenarios, compensation payments for SNGLs and the number of beef cattle had a stronger influence on the use of SNGLs; however, a further increase in the payment level above current levels increased windfall effects and worsened inefficient labour allocation, as the labour shortage is the main bottleneck. The relatively high profitability of mulching, the labour shortage, and the moderate profitability of beef production are all factors that hamper the use of SNGL. As for the impact on regional economic profitability, the increased use of SNGLs caused crowding-out effects and lowered the regional market-based GM due to the relatively high costs of managing SNGLs. Nonetheless, compensation payments contribute to the overall income level of farm holdings in the region. Overall, intervening in beef production levels might be more effective in increasing the use of SNGLs given its smaller negative impacts on economic profitability, but should be accompanied by cost-effective and labour-efficient management practices, which will eventually lead to increasing profitability of beef production. Such measures will also promote the alternative use of products, such as bioenergy production.

## Supplementary Information

Supplementary material 1

Supplementary material 2

## Data Availability

All data supporting the findings of this study are available in/from the Estonian nature information system (Estonian Environment Agency), the Estonian Semi-Natural Community Conservation Association and the Register of Agricultural Support and Agricultural Parcels (Agricultural Register and Information Board), an Estonian farm data handbook (Agricultural Research Centre in Estonia), Statistics Estonia (https://www.stat.ee), FEEDBASE (https://www.feedbase.ch/), and an agricultural handbook of Brandenburg in Germany (MLUK).
