# Supplementary material for: Modelling Alternative Economic Incentive Schemes for Semi-Natural Grassland Conservation in Estonia
Source: Environ Manage. 2024 Aug 1;74(4):757–74. doi: 10.1007/s00267-024-02011-2 (PMC11393159; doi:10.1007/s00267-024-02011-2)
Supplement: Supplementary file 1 — Supplementary material 1 [file 267_2024_2011_MOESM1_ESM.docx]

Supplementary material 1: Modelled parameters used in the bio-farm economic model and data sources

**Modelling Alternative Economic Incentive Schemes for Semi-Natural Grassland Conservation in Estonia**

# 1. Field activities

## Yield

| Land use type | Field activity | yield(t/ha) | Source |
| --- | --- | --- | --- |
| Semi-natural grasslands | Grazing | 1.2 | Piirsalu et al. (2019) |
|  | Mowing/1 cut | 1.9 | Piirsalu et al. (2019) |
|  | Biomass production | 1.9 | Piirsalu et al. (2019) |
|  | Mulching | 0 | Piirsalu et al. (2019) |
| Permanent grassland | Grazing | 3.0 | Agricultural Research Centre (2021) |
|  | Mowing/1 cut | 3.4 | Own calculation based on Agricultural Research Centre (2021) and MULK (2021) |
|  | Mowing/2 cut | 6.0 | Agricultural Research Centre (2021) |
|  | Mowing/3 cut | 9.7 | Own calculation based on Agricultural Research Centre (2021) and MULK (2021) |
|  | Mixed (Mowing and grazing) | 5.1 | Own calculation based on Agricultural Research Centre (2021) and MULK (2021) |
|  | Mixed (Silage production and grazing) | 6.4 | Own calculation based on Agricultural Research Centre (2021) and MULK (2021) |
|  | Mulching | 0 |  |
| Arable land | Field grass for silage | 5.3 | Agricultural Research Centre (2021) |
|  | Field grass for silage-baling | 5.3 | Agricultural Research Centre (2021) |
|  | Mechanical tillage | 0 |  |
|  | Field beans/ploughing | 2.5 | Agricultural Research Centre (2021) |
|  | Field beans/reduced tillage | 2.5 | Agricultural Research Centre (2021) |
|  | Field beans/direct seeding | 2.5 | Agricultural Research Centre (2021) |
|  | Field peas/ploughing | 2.0 | Agricultural Research Centre (2021) |
|  | Field peas/reduced tillage | 2.0 | Agricultural Research Centre (2021) |
|  | Field peas/direct seeding | 2.0 | Agricultural Research Centre (2021) |
|  | Green maize/ploughing | 10.6 | Agricultural Research Centre (2021) |
|  | Oats/ploughing | 3.0 | Agricultural Research Centre (2021) |
|  | Oats/reduced tillage | 3.0 | Agricultural Research Centre (2021) |
|  | Oats/direct seeding | 3.0 | Agricultural Research Centre (2021) |
|  | Rye/ploughing | 4.5 | Agricultural Research Centre (2021) |
|  | Rye/reduced tillage | 4.5 | Agricultural Research Centre (2021) |
|  | Rye/direct seeding | 4.5 | Agricultural Research Centre (2021) |
|  | Spring barley/ploughing | 3.0 | Agricultural Research Centre (2021) |
|  | Spring barley/reduced tillage | 3.0 | Agricultural Research Centre (2021) |
|  | Spring barley/direct seeding | 3.0 | Agricultural Research Centre (2021) |
|  | Spring wheat/ploughing | 3.0 | Agricultural Research Centre (2021) |
|  | Spring wheat/ reduced tillage | 3.0 | Agricultural Research Centre (2021) |
|  | Spring wheat/ direct seeding | 3.0 | Agricultural Research Centre (2021) |
|  | Winter wheat/ploughing | 5.0 | Agricultural Research Centre (2021) |
|  | Winter wheat/reduced tillage | 5.0 | Agricultural Research Centre (2021) |
|  | Winter wheat/direct seeding | 5.0 | Agricultural Research Centre (2021) |
|  | Buckwheat/ploughing | 1.0 | Agricultural Research Centre (2021) |
|  | Buckwheat/reduced tillage | 1.0 | Agricultural Research Centre (2021) |
|  | Buckwheat/direct seeding | 1.0 | Agricultural Research Centre (2021) |
|  | Potatoes/ploughing | 20.0 | Agricultural Research Centre (2021) |
|  | Spring rapeseed/ploughing | 2.0 | Agricultural Research Centre (2021) |
|  | Winter rapeseed/ploughing | 2.0 | Agricultural Research Centre (2021) |

## Price

| Land use type | Field activity | Price (€/t) | Source |
| --- | --- | --- | --- |
| Semi-natural grasslands | Grazing | 0 |  |
|  | Mowing/1 cut | 70 | (Price of hay for export) Personal communication with experts |
|  | Biomass production | 70 | (Price of hay for biomass heating plants) Personal communication with experts |
|  | Mulching | 0 |  |
| Permanent grassland | Grazing | 0 |  |
|  | Mowing/1 cut | 70 | (Price of hay for export) Personal communication with experts |
|  | Mowing/2 cut | 70 | (Price of hay for export) Personal communication with experts |
|  | Mowing/3 cut | 70 | (Price of hay for export) Personal communication with experts |
|  | Mixed (Mowing and grazing) | 70 | (Price of hay for export) Personal communication with experts |
|  | Mixed (Silage production and grazing) | 0 |  |
|  | Mulching | 0 |  |
| Arable land | Field grass for silage | 0 |  |
|  | Field grass for silage-baling | 0 |  |
|  | Mechanical tillage | 0 |  |
|  | Field beans/ploughing | 236 | Agricultural Research Centre (2021) |
|  | Field beans/reduced tillage | 236 | Agricultural Research Centre (2021) |
|  | Field beans/direct seeding | 236 | Agricultural Research Centre (2021) |
|  | Field peas/ploughing | 201 | Agricultural Research Centre (2021) |
|  | Field peas/reduced tillage | 201 | Agricultural Research Centre (2021) |
|  | Field peas/direct seeding | 201 | Agricultural Research Centre (2021) |
|  | Green maize/ploughing | 64 | Agricultural Research Centre (2021) |
|  | Oats/ploughing | 131 | Agricultural Research Centre (2021) |
|  | Oats/reduced tillage | 131 | Agricultural Research Centre (2021) |
|  | Oats/direct seeding | 131 | Agricultural Research Centre (2021) |
|  | Rye/ploughing | 133 | Agricultural Research Centre (2021) |
|  | Rye/reduced tillage | 133 | Agricultural Research Centre (2021) |
|  | Rye/direct seeding | 133 | Agricultural Research Centre (2021) |
|  | Spring barley/ploughing | 160 | Agricultural Research Centre (2021) |
|  | Spring barley/reduced tillage | 160 | Agricultural Research Centre (2021) |
|  | Spring barley/direct seeding | 160 | Agricultural Research Centre (2021) |
|  | Spring wheat/ploughing | 170 | Agricultural Research Centre (2021) |
|  | Spring wheat/ reduced tillage | 170 | Agricultural Research Centre (2021) |
|  | Spring wheat/ direct seeding | 170 | Agricultural Research Centre (2021) |
|  | Winter wheat/ploughing | 170 | Agricultural Research Centre (2021) |
|  | Winter wheat/reduced tillage | 170 | Agricultural Research Centre (2021) |
|  | Winter wheat/direct seeding | 170 | Agricultural Research Centre (2021) |
|  | Buckwheat/ploughing | 343 | Agricultural Research Centre (2021) |
|  | Buckwheat/reduced tillage | 343 | Agricultural Research Centre (2021) |
|  | Buckwheat/direct seeding | 343 | Agricultural Research Centre (2021) |
|  | Potatoes/ploughing | 121 | Agricultural Research Centre (2021) |
|  | Spring rapeseed/ploughing | 415 | Agricultural Research Centre (2021) |
|  | Winter rapeseed/ploughing | 415 | Agricultural Research Centre (2021) |

## Variable cost

| Land use type | Field activity | Variable cost (€/ha) | Additional variable cost* (€/ha) | Source |
| --- | --- | --- | --- | --- |
| Semi-natural grasslands | Grazing | 70 | 130 | Own calculation based on Agricultural Research Centre (2021) and MULK (2021), own estimation of the additional variable cost |
|  | Mowing/1 cut | 131 | 130 | Own calculation based on Agricultural Research Centre (2021), own estimation of the additional variable cost |
|  | Biomass production | 131 | 130 | Assumed same as mowing/1 cut on semi-natural grassland, own estimation of the additional variable cost |
|  | Mulching | 51 | 130 | Own calculation based on Agricultural Research Centre (2021), own estimation of the additional variable cost |
| Permanent grassland | Grazing | 161 | 0 | Agricultural Research Centre (2021) |
|  | Mowing/1 cut | 188 | 0 | Own calculation based on Agricultural Research Centre (2021) and MULK (2021) |
|  | Mowing/2 cut | 335 | 0 | Agricultural Research Centre (2021) |
|  | Mowing/3 cut | 539 | 0 | Own calculation based on Agricultural Research Centre (2021) and MULK (2021) |
|  | Mixed (Mowing and grazing) | 254 | 0 | Own calculation based on Agricultural Research Centre (2021) |
|  | Mixed (Silage production and grazing) | 222 | 0 | Own calculation based on Agricultural Research Centre (2021) |
|  | Mulching | 53 | 0 | Own calculation based on Agricultural Research Centre (2021) |
| Arable land | Field grass for silage | 286 | 0 | Agricultural Research Centre (2021) |
|  | Field grass for silage-baling | 409 | 0 | Agricultural Research Centre (2021) |
|  | Mechanical tillage | 420 | 0 | Agricultural Research Centre (2021) |
|  | Field beans/ploughing | 727 | 0 | Agricultural Research Centre (2021) |
|  | Field beans/reduced tillage | 702 | 0 | Agricultural Research Centre (2021) |
|  | Field beans/direct seeding | 644 | 0 | Agricultural Research Centre (2021) |
|  | Field peas/ploughing | 629 | 0 | Agricultural Research Centre (2021) |
|  | Field peas/reduced tillage | 603 | 0 | Agricultural Research Centre (2021) |
|  | Field peas/direct seeding | 455 | 0 | Agricultural Research Centre (2021) |
|  | Green maize/ploughing | 911 | 0 | Agricultural Research Centre (2021) |
|  | Oats/ploughing | 566 | 0 | Agricultural Research Centre (2021) |
|  | Oats/reduced tillage | 540 | 0 | Agricultural Research Centre (2021) |
|  | Oats/direct seeding | 484 | 0 | Agricultural Research Centre (2021) |
|  | Rye/ploughing | 823 | 0 | Agricultural Research Centre (2021) |
|  | Rye/reduced tillage | 793 | 0 | Agricultural Research Centre (2021) |
|  | Rye/direct seeding | 740 | 0 | Agricultural Research Centre (2021) |
|  | Spring barley/ploughing | 563 | 0 | Agricultural Research Centre (2021) |
|  | Spring barley/reduced tillage | 538 | 0 | Agricultural Research Centre (2021) |
|  | Spring barley/direct seeding | 481 | 0 | Agricultural Research Centre (2021) |
|  | Spring wheat/ploughing | 678 | 0 | Agricultural Research Centre (2021) |
|  | Spring wheat/ reduced tillage | 652 | 0 | Agricultural Research Centre (2021) |
|  | Spring wheat/ direct seeding | 596 | 0 | Agricultural Research Centre (2021) |
|  | Winter wheat/ploughing | 842 | 0 | Agricultural Research Centre (2021) |
|  | Winter wheat/reduced tillage | 813 | 0 | Agricultural Research Centre (2021) |
|  | Winter wheat/direct seeding | 760 | 0 | Agricultural Research Centre (2021) |
|  | Buckwheat/ploughing | 434 | 0 | Agricultural Research Centre (2021) |
|  | Buckwheat/reduced tillage | 383 | 0 | Agricultural Research Centre (2021) |
|  | Buckwheat/direct seeding | 343 | 0 | Agricultural Research Centre (2021) |
|  | Potatoes/ploughing | 3424 | 0 | Agricultural Research Centre (2021) |
|  | Spring rapeseed/ploughing | 768 | 0 | Agricultural Research Centre (2021) |
|  | Winter rapeseed/ploughing | 718 | 0 | Agricultural Research Centre (2021) |

* Additional machinery and fuel costs for travel and transport to and from farmsteads to SNGL based on expert judgements

## Agri-environmental payments and compensation payments

| Land use type | Field activity | Direct payments (€/ha) | Compensation payments (€/ha) | Source |
| --- | --- | --- | --- | --- |
| Semi-natural grasslands | Grazing | 160 | 150 | Agricultural Research Centre (2021) and the Estonia Environmental Board (2021) |
|  | Mowing/1 cut | 160 | 80 | Agricultural Research Centre (2021) and the Estonia Environmental Board (2021) |
|  | Biomass production | 160 | 80 | Agricultural Research Centre (2021) and the Estonia Environmental Board (2021) |
|  | Mulching | 160 | 0 | Agricultural Research Centre (2021) |
| Permanent grassland | Grazing | 160 | 0 | Agricultural Research Centre (2021) |
|  | Mowing/1 cut | 160 | 0 | Agricultural Research Centre (2021) |
|  | Mowing/2 cut | 160 | 0 | Agricultural Research Centre (2021) |
|  | Mowing/3 cut | 160 | 0 | Agricultural Research Centre (2021) |
|  | Mixed (Mowing and grazing) | 160 | 0 | Agricultural Research Centre (2021) |
|  | Mixed (Silage production and grazing) | 160 | 0 | Agricultural Research Centre (2021) |
|  | Mulching | 160 | 0 | Agricultural Research Centre (2021) |
| Arable land | Field grass for silage | 210 | 0 | Agricultural Research Centre (2021) and the inventory of the Register of Agricultural Support and Agricultural Parcels (2022) |
|  | Field grass for silage-baling | 210 | 0 | Agricultural Research Centre (2021) and the inventory of the Register of Agricultural Support and Agricultural Parcels (2022) |
|  | Mechanical tillage | 160 | 0 | Agricultural Research Centre (2021) and the inventory of the Register of Agricultural Support and Agricultural Parcels (2022) |
|  | Field beans/ploughing | 210 | 0 | Agricultural Research Centre (2021) and the inventory of the Register of Agricultural Support and Agricultural Parcels (2022) |
|  | Field beans/reduced tillage | 210 | 0 | Agricultural Research Centre (2021) and the inventory of the Register of Agricultural Support and Agricultural Parcels (2022) |
|  | Field beans/direct seeding | 210 | 0 | Agricultural Research Centre (2021) and the inventory of the Register of Agricultural Support and Agricultural Parcels (2022) |
|  | Field peas/ploughing | 210 | 0 | Agricultural Research Centre (2021) and the inventory of the Register of Agricultural Support and Agricultural Parcels (2022) |
|  | Field peas/reduced tillage | 210 | 0 | Agricultural Research Centre (2021) and the inventory of the Register of Agricultural Support and Agricultural Parcels (2022) |
|  | Field peas/direct seeding | 210 | 0 | Agricultural Research Centre (2021) and the inventory of the Register of Agricultural Support and Agricultural Parcels (2022) |
|  | Green maize/ploughing | 210 | 0 | Agricultural Research Centre (2021) and the inventory of the Register of Agricultural Support and Agricultural Parcels (2022) |
|  | Oats/ploughing | 210 | 0 | Agricultural Research Centre (2021) and the inventory of the Register of Agricultural Support and Agricultural Parcels (2022) |
|  | Oats/reduced tillage | 210 | 0 | Agricultural Research Centre (2021) and the inventory of the Register of Agricultural Support and Agricultural Parcels (2022) |
|  | Oats/direct seeding | 210 | 0 | Agricultural Research Centre (2021) and the inventory of the Register of Agricultural Support and Agricultural Parcels (2022) |
|  | Rye/ploughing | 210 | 0 | Agricultural Research Centre (2021) and the inventory of the Register of Agricultural Support and Agricultural Parcels (2022) |
|  | Rye/reduced tillage | 210 | 0 | Agricultural Research Centre (2021) and the inventory of the Register of Agricultural Support and Agricultural Parcels (2022) |
|  | Rye/direct seeding | 210 | 0 | Agricultural Research Centre (2021) and the inventory of the Register of Agricultural Support and Agricultural Parcels (2022) |
|  | Spring barley/ploughing | 210 | 0 | Agricultural Research Centre (2021) and the inventory of the Register of Agricultural Support and Agricultural Parcels (2022) |
|  | Spring barley/reduced tillage | 210 | 0 | Agricultural Research Centre (2021) and the inventory of the Register of Agricultural Support and Agricultural Parcels (2022) |
|  | Spring barley/direct seeding | 210 | 0 | Agricultural Research Centre (2021) and the inventory of the Register of Agricultural Support and Agricultural Parcels (2022) |
|  | Spring wheat/ploughing | 210 | 0 | Agricultural Research Centre (2021) and the inventory of the Register of Agricultural Support and Agricultural Parcels (2022) |
|  | Spring wheat/ direct seeding | 210 | 0 | Agricultural Research Centre (2021) and the inventory of the Register of Agricultural Support and Agricultural Parcels (2022) |
|  | Winter wheat/ploughing | 210 | 0 | Agricultural Research Centre (2021) and the inventory of the Register of Agricultural Support and Agricultural Parcels (2022) |
|  | Winter wheat/ploughing | 210 | 0 | Agricultural Research Centre (2021) and the inventory of the Register of Agricultural Support and Agricultural Parcels (2022) |
|  | Winter wheat/reduced tillage | 210 | 0 | Agricultural Research Centre (2021) and the inventory of the Register of Agricultural Support and Agricultural Parcels (2022) |
|  | Winter wheat/direct seeding | 210 | 0 | Agricultural Research Centre (2021) and the inventory of the Register of Agricultural Support and Agricultural Parcels (2022) |
|  | Buckwheat/ploughing | 210 | 0 | Agricultural Research Centre (2021) and the inventory of the Register of Agricultural Support and Agricultural Parcels (2022) |
|  | Buckwheat/reduced tillage | 210 | 0 | Agricultural Research Centre (2021) and the inventory of the Register of Agricultural Support and Agricultural Parcels (2022) |
|  | Buckwheat/direct seeding | 210 | 0 | Agricultural Research Centre (2021) and the inventory of the Register of Agricultural Support and Agricultural Parcels (2022) |
|  | Potatoes/ploughing | 210 | 0 | Agricultural Research Centre (2021) and the inventory of the Register of Agricultural Support and Agricultural Parcels (2022) |
|  | Spring rapeseed/ploughing | 210 | 0 | Agricultural Research Centre (2021) and the inventory of the Register of Agricultural Support and Agricultural Parcels (2022) |
|  | Winter rapeseed/ploughing | 210 | 0 | Agricultural Research Centre (2021) and the inventory of the Register of Agricultural Support and Agricultural Parcels (2022) |

## Labour requirement

| Land use type | Field activity | Labour demand (h/ha) | Additional labour demand* (h/ha) | Source |
| --- | --- | --- | --- | --- |
| Semi-natural grasslands | Grazing | 1.9 | 1.0 | MULK (2021), own estimation of the additional labour demand |
|  | Mowing/1 cut | 2.4 | 1.0 | MULK (2021), own estimation of the additional labour demand |
|  | Biomass production | 2.4 | 1.0 | Assumed same as mowing/1 cut on semi-natural grassland |
|  | Mulching | 0.6 | 1.0 | Own calculation based on MULK (2021), own estimation of the additional labour demand |
| Permanent grassland | Grazing | 2.7 | 0 | MULK (2021) |
|  | Mowing/1 cut | 2.4 | 0 | MULK (2021) |
|  | Mowing/2 cut | 5.0 | 0 | MULK (2021) |
|  | Mowing/3 cut | 6.5 | 0 | MULK (2021) |
|  | Mixed (Mowing and grazing) | 3.7 | 0 | Own calculation based on MULK (2021) |
|  | Mixed (Silage production and grazing) | 4.0 | 0 | Own calculation based on MULK (2021) |
|  | Mulching | 0.6 | 0 | MULK (2021) |
| Arable land | Field grass for silage | 4.1 | 0 | Own calculation based on MULK (2021) |
|  | Field grass for silage-baling | 5.9 | 0 | Own calculation based on MULK (2021) |
|  | Mechanical tillage | 4.8 | 0 | MULK (2021) |
|  | Field beans/ploughing | 2.4 | 0 | MULK (2021) |
|  | Field beans/reduced tillage | 2.4 | 0 | Own calculation based on MULK (2021) |
|  | Field beans/direct seeding | 1.7 | 0 | Own calculation based on MULK (2021) |
|  | Field peas/ploughing | 2.4 | 0 | MULK (2021) |
|  | Field peas/reduced tillage | 2.4 | 0 | Own calculation based on MULK (2021) |
|  | Field peas/direct seeding | 1.7 | 0 | Own calculation based on MULK (2021) |
|  | Green maize/ploughing | 4.3 | 0 | MULK (2021) |
|  | Oats/ploughing | 2.3 | 0 | MULK (2021) |
|  | Oats/reduced tillage | 2.3 | 0 | Own calculation based on MULK (2021) |
|  | Oats/direct seeding | 1.6 | 0 | Own calculation based on MULK (2021) |
|  | Rye/ploughing | 2.5 | 0 | MULK (2021) |
|  | Rye/reduced tillage | 2.5 | 0 | Own calculation based on MULK (2021) |
|  | Rye/direct seeding | 1.8 | 0 | Own calculation based on MULK (2021) |
|  | Spring barley/ploughing | 2.4 | 0 | MULK (2021) |
|  | Spring barley/reduced tillage | 2.4 | 0 | Own calculation based on MULK (2021) |
|  | Spring barley/direct seeding | 1.7 | 0 | Own calculation based on MULK (2021) |
|  | Spring wheat/ploughing | 2.4 | 0 | MULK (2021) |
|  | Spring wheat/ploughing | 2.4 | 0 | MULK (2021) |
|  | Spring wheat/ direct seeding | 1.7 | 0 | Own calculation based on MULK (2021) |
|  | Winter wheat/ploughing | 2.7 | 0 | Own calculation based on MULK (2021) |
|  | Winter wheat/reduced tillage | 2.6 | 0 | Own calculation based on MULK (2021) |
|  | Winter wheat/direct seeding | 1.9 | 0 | Own calculation based on MULK (2021) |
|  | Buckwheat/ploughing | 2.3 | 0 | Assumed the same as oats |
|  | Buckwheat/reduced tillage | 2.3 | 0 | Assumed the same as oats |
|  | Buckwheat/direct seeding | 1.6 | 0 | Assumed the same as oats |
|  | Potatoes/ploughing | 15.2 | 0 | MULK (2021) |
|  | Spring rapeseed/ploughing | 2.5 | 0 | Own calculation based on MULK (2021) |
|  | Winter rapeseed/ploughing | 2.4 | 0 | Own calculation based on MULK (2021) |

*The labour demand of travel and transport to and from farms to semi-natural grasslands

## Feed nutritional values

| Land use type | Field activity | Net energy lactose (MJ/kg of DM*) | Crude-protein  (kg/kg of DM | Crude-fibre  (kg/kg of DM | Source |
| --- | --- | --- | --- | --- | --- |
| Semi-natural grasslands | Grazing | 5.46 | 0.16 | 0.24 | Piirsalu et al. (2019) |
|  | Mowing/1 cut | 5.40 | 0.10 | 0.23 | Piirsalu et al. (2019) |
|  | Biomass production | - | - | - |  |
|  | Mulching | - | - | - |  |
| Permanent grassland | Grazing | 5.77 | 0.14 | 0.24 | FEEDBASE (Agroscope, et al., n.d.) |
|  | Mowing/1 cut | 5.31 | 0.13 | 0.26 | FEEDBASE (Agroscope, et al., n.d.) |
|  | Mowing/2 cut | 5.52 | 0.15 | 0.24 | FEEDBASE (Agroscope, et al., n.d.) |
|  | Mowing/3 cut | 5.84 | 0.18 | 0.22 | FEEDBASE (Agroscope, et al., n.d.) |
|  | Mixed (Mowing and grazing) | 5.71 | 0.16 | 0.23 | FEEDBASE (Agroscope, et al., n.d.) |
|  | Mixed (Silage production and grazing) | 5.90 | 0.17 | 0.23 | FEEDBASE (Agroscope, et al., n.d.) |
|  | Mulching | 0.0 | 0.0 | 0.0 | FEEDBASE (Agroscope, et al., n.d.) |
| Arable land | Field grass for silage | 6.26 | 0.24 | 0.17 | FEEDBASE (Agroscope, et al., n.d.) |
|  | Field grass for silage-baling | 6.26 | 0.24 | 0.17 | FEEDBASE (Agroscope, et al., n.d.) |
|  | Mechanical tillage | - | - | - |  |
|  | Field beans/ploughing | 7.74 | 0.26 | 0.05 | FEEDBASE (Agroscope, et al., n.d.) |
|  | Field beans/reduced tillage | 7.74 | 0.26 | 0.05 | FEEDBASE (Agroscope, et al., n.d.) |
|  | Field beans/direct seeding | 7.74 | 0.26 | 0.05 | FEEDBASE (Agroscope, et al., n.d.) |
|  | Field peas/ploughing | 8.09 | 0.21 | 0.05 | FEEDBASE (Agroscope, et al., n.d.) |
|  | Field peas/reduced tillage | 8.09 | 0.21 | 0.05 | FEEDBASE (Agroscope, et al., n.d.) |
|  | Field peas/direct seeding | 8.09 | 0.21 | 0.05 | FEEDBASE (Agroscope, et al., n.d.) |
|  | Green maize/ploughing | 6.63 | 0.07 | 0.02 | FEEDBASE (Agroscope, et al., n.d.) |
|  | Oats/ploughing | 8.20 | 0.13 | 0.09 | FEEDBASE (Agroscope, et al., n.d.) |
|  | Oats/reduced tillage | 8.20 | 0.13 | 0.09 | FEEDBASE (Agroscope, et al., n.d.) |
|  | Oats/direct seeding | 8.20 | 0.13 | 0.09 | FEEDBASE (Agroscope, et al., n.d.) |
|  | Rye/ploughing | 8.27 | 0.12 | 0.02 | FEEDBASE (Agroscope, et al., n.d.) |
|  | Rye/reduced tillage | 8.27 | 0.12 | 0.02 | FEEDBASE (Agroscope, et al., n.d.) |
|  | Rye/direct seeding | 8.27 | 0.12 | 0.02 | FEEDBASE (Agroscope, et al., n.d.) |
|  | Spring barley/ploughing | 8.63 | 0.11 | 0.08 | FEEDBASE (Agroscope, et al., n.d.) |
|  | Spring barley/reduced tillage | 8.63 | 0.11 | 0.08 | FEEDBASE (Agroscope, et al., n.d.) |
|  | Spring barley/direct seeding | 8.63 | 0.11 | 0.08 | FEEDBASE (Agroscope, et al., n.d.) |
|  | Spring wheat/ploughing | 8.76 | 0.14 | 0.02 | FEEDBASE (Agroscope, et al., n.d.) |
|  | Spring wheat/ reduced tillage | 8.76 | 0.14 | 0.02 | FEEDBASE (Agroscope, et al., n.d.) |
|  | Spring wheat/ direct seeding | 8.76 | 0.14 | 0.02 | FEEDBASE (Agroscope, et al., n.d.) |
|  | Winter wheat/ploughing | 8.76 | 0.14 | 0.02 | FEEDBASE (Agroscope, et al., n.d.) |
|  | Winter wheat/reduced tillage | 8.76 | 0.14 | 0.02 | FEEDBASE (Agroscope, et al., n.d.) |
|  | Winter wheat/direct seeding | 8.76 | 0.14 | 0.02 | FEEDBASE (Agroscope, et al., n.d.) |
|  | Buckwheat/ploughing | - | - | - |  |
|  | Buckwheat/reduced tillage | - | - | - |  |
|  | Buckwheat/direct seeding | - | - | - |  |
|  | Potatoes/ploughing | - | - | - |  |
|  | Spring rapeseed/ploughing | - | - | - |  |
|  | Winter rapeseed/ploughing | - | - | - |  |

*DM-dry matter

# 2. Livestock activities

| Livestock type | GM (€/LU*) | Labour demand (h/LU) | Maximum dry matter intake (kg/year) | Minimum net energy for lactation intake (MJ/year) | Minimum crude-protein intake (kg/year) | Minimum crude-fibre intake (kg/year) |
| --- | --- | --- | --- | --- | --- | --- |
| Dairy cow | 2,867 | 42 | 6,570 | 41,829 | 949 | 1,314 |
| Dairy cow offspring | -611 | -** | 4,562 | 17,155 | 438 | 876 |
| Beef cattle | 506 | 15 | 5,110 | 29,200 | 686 | 1,022 |
| Beef cattleoffspring | -281 | -** | 2,844 | 13,176 | 343 | 518 |
| Data source | MULK (2021) | MULK (2021) | DLG (1997) | DLG (1997) | DLG (1997) | DLG (1997) |

*LU…livestock unit **labour demand for offspring is included in the labour demand of the adult cows

# References

Agroscope, Swiss National Science Foundation, & University of Zürich. (n.d.). *FEEDBASE (The Swiss Feed Database)*. https://www.feedbase.ch/

ARC Agricultural Research Centre [Põllumajandusuuringute Keskus]. (2021). *Kattetulu arvestused taime- ja loomakasvatuses 2021 [Accounting for the margin in crop and livestock production 2021]*. https://pmk.agri.ee/sites/default/files/inline-files/2021_kattetulu_220303.pdf

DLG. (1997). Futterwerttabellen Wiederkäuer. *DLG-Verlag: Frankfurt [M]*.

MULK Ministry of Agriculture, E. and C. P. (2021). *Datensammlung für die betriebswirtschaftliche Bewertung landwirtschaftlicher Produktionsverfahren im Land Brandenburg [Data collection for the economic evaluation of agricultural production methods in the state of Brandenburg]*.

Piirsalu, P., Arney, D., Kass, M., Leming, R., Ots, M., & Tölp, S. (2019). Aastaringselt välitingimustes peetavate lihaveiste ja lammaste tervise- ning heaolunäitajad . Lihaveiste ja lammaste heaoluindikaatorite väljatöötamine . Poollooduslikud kooslused lihaveiste ja lammaste söödabaasina , soovitused lisasöötmise vajalikkuse [. In *Institute of Veterinary Medicine and Animal Husbandry, Estonian University of Life Sciences*. https://www.pikk.ee/wp-content/uploads/2021/04/Lopparuanne_Aaastaringselt_pikk-002.pdf

PRIA Public of Estonia Agricultural Registers and Information Board. (n.d.). *Support for environmentally friendly management in 2022*. Retrieved October 13, 2023, from https://www.pria.ee/toetused/KSM_2022

Public of Estonia Environmental Board. (2021). *ACTION PLAN FOR SEMI-NATURAL GRASSLANDS*. https://keskkonnaamet.ee/en/media/5321/download
